# *In Ovo* Monitoring of Smooth Muscle Fiber Development in the Chick Embryo: Diffusion Tensor Imaging with Histologic Correlation

**DOI:** 10.1371/journal.pone.0034009

**Published:** 2012-03-23

**Authors:** Jianrong Xu, Zachary DelProposto, Zien Zhou, Huicong Shen, Stephanie Yang Xuan, Qing Hang Li, E. Mark Haacke, Jiani Hu

**Affiliations:** 1 Department of Radiology, Renji Hospital, Shanghai Jiaotong University School of Medicine, Shanghai, China; 2 Department of Radiology, Henry Ford Hospital, Detroit, Michigan, United States of America; 3 Department of Radiology, Beijing Tiantan Hospital, Capital Medical University, Beijing, China; 4 Faculty of Arts and Science, University of Toronto, Toronto, Ontario, Canada; 5 Department of Neurosurgery, Wayne State University, Detroit, Michigan, United States of America; 6 Department of Radiology, Wayne State University, Detroit, Michigan, United States of America; Universitat Pompeu Fabra, Spain

## Abstract

**Background:**

Magnetic resonance imaging is a noninvasive method of evaluating embryonic development. Magnetic resonance diffusion tensor imaging, which is based on the measuring the directional diffusivity of water molecules, is an established method of evaluating tissue structure. Prolonged imaging times have precluded the use of embryonic diffusion tensor imaging due to motion artifact. Using temperature-based motion suppression, we aimed to investigate whether diffusion tensor imaging can be used to monitor embryonic smooth muscle development *in ovo*, and to determine the correlation between histologically-derived muscle fiber fraction, day of incubation and diffusion tensor imaging fractional anisotropy values and length of tracked fibers.

**Methodology/Principal Findings:**

From a set of 82 normally developing fertile chicken eggs, 5 eggs were randomly chosen each day from incubation days 5 to 18 and cooled using a dual-cooling technique prior to and during magnetic resonance imaging at 3.0 Tesla. Smooth muscle fibers of the gizzard were tracked using region of interests placed over the gizzard. Following imaging, the egg was cracked and the embryo was fixated and sectioned, and a micrograph most closely corresponding to the acquired magnetic resonance image was made. Smooth muscle fiber fraction was determined using an automated computer algorithm.

**Conclusions/Significance:**

We show that diffusion tensor images of smooth muscle within the embryonic gizzard can be acquired in ovo from incubation day 11 through hatching. Length of tracked fibers and day of incubation were found to have statistical significance (p<0.05) by multiple linear regression correlation with histologic specimens of sacrificed embryos from day 11 of incubation through hatching. The morphologic pattern of development in our histologic specimens corresponds to the development of embryonic gizzard as reported in the literature. These results suggest that diffusion tensor imaging can provide a noninvasive method of evaluating *in ovo* development of smooth muscle tissue.

## Introduction

Animal models are essential for understanding embryonic development and disease pathogenesis from the anatomic to the molecular level. *In vivo* evaluation of tissue structure improves the understanding of tissue differentiation within the developmental environment [1]. The chick embryo is an animal model which is highly accessible and economical; excepting oxygen and heat, it includes all necessary constituents for development [2]. Magnetic resonance (MR) imaging is a commonly used method for *in vivo* anatomic imaging, and has been used to image avian embryos. With the refinement of advanced MR techniques such as magnetic resonance spectroscopy, blood-oxygen level dependent imaging, and diffusion weighted imaging, anatomic data can be augmented with metabolic, physiologic, and structural information [3], [4]. The inherent low signal-to-noise ratio (SNR) in MR imaging prolongs imaging time, especially with advanced techniques, and the acquisition of high quality images is particularly challenged or even precluded by subject motion [4]–[6]. Embryonic anesthesia is one method of suppressing bulk motion artifact permitting real-time cardiac imaging to be performed [4], [6], [7]. Embryonic cooling is another method that has recently been proven effective for the acquisition of high-quality *in ovo* anatomic images of chick embryos [5].

Harnessing the effects of Brownian motion, diffusion weighted imaging (DWI) translates the restrictive effect of tissue structure on water molecule mobility into visible signal intensity differences [8]. Extending this concept, diffusion tensor imaging (DTI) measures the preferred diffusivity direction (the displacement distribution) in addition to bulk water diffusivity [8]. Displacement distribution measurement provides a method of evaluating tissue structure and organization, and diffusion tensor imaging is an established method of studying nerve and muscle tissue microstructure [9]. A disadvantage of DTI is the prolonged imaging duration and resultant degradation of image data due to subject motion. The purpose of this study is to determine whether diffusion tensor imaging can be used to monitor embryonic smooth muscle development *in ovo* using embryonic cooling, and investigate whether serial DTI smooth muscle tractography correlates with *ex vivo* histology.

## Results

**Figure 1:**
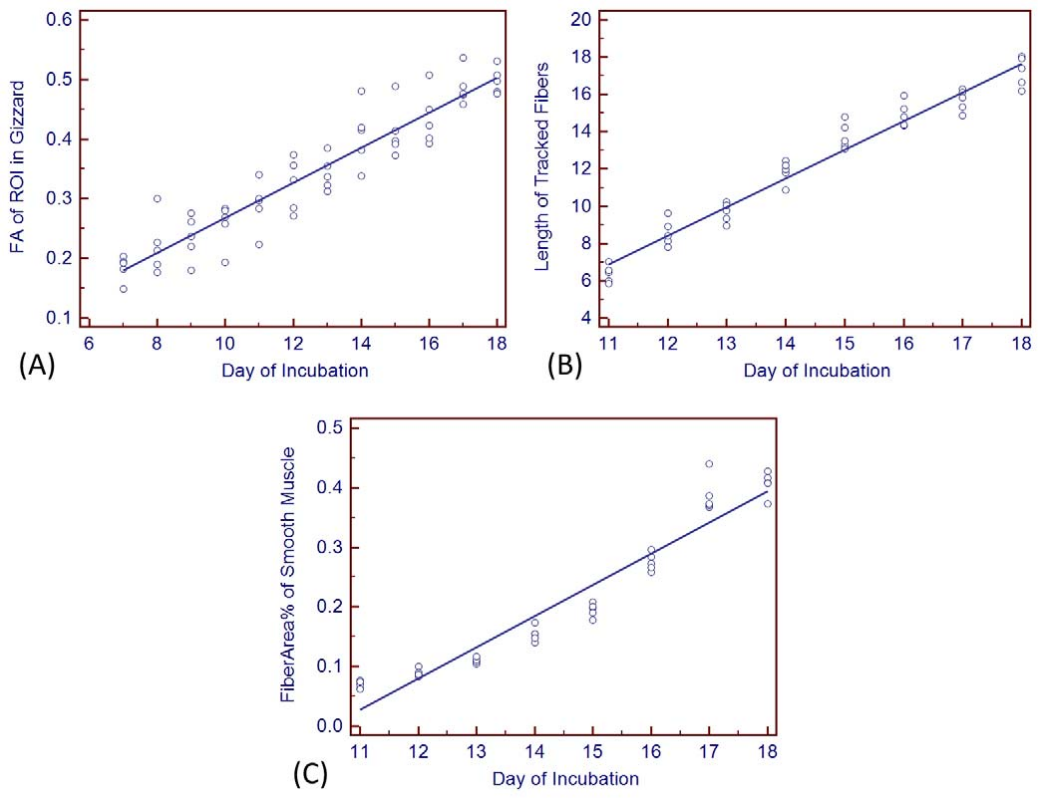
Scatter plots of measured smooth muscle parameters vs. incubation day. (**A**) Relationship between DTI-derived fractional anisotropy (FA) and incubation day. (**B**) Relationship between histology-derived FiberArea% and incubation day. (**C**) Relationship between length fibers tracked with DTI and incubation day. For all figures, the trend line indicates the result of linear regression analysis.

The proventriculus (primitive gizzard) could be discriminated on T2 weighted images from day 7. Beginning with day 11, muscle fibers of the gizzard could be tracked with DTI and the fiber length derived. As shown in Table 1 and Figs. 1(A) and 1(B), fractional anisotropy (FA) values and fiber length show a progressive linear increase with each successive day of incubation. Chick embryo muscle fibers could not be adequately evaluated by light microscopy until day 10 or 11, the point at which individual muscle fiber bundles could be visually discerned. Subsequent days showed a progressive linear increase in trabecular pattern as quantified by FiberArea%, and are shown in Table 1 and Fig. 1(C).

**Table 1:** Smooth muscle fractional anisotropy, tracked fiber length and FiberArea% values for chick embryonic gizzard at each day of incubation.

Incubation Day	Fractional Anisotropy (FA) ± s.d.	Length± s.d. (mm)	FiberArea% ± s.d.
Day 5	†	‡	##‡
Day 6	†	‡	‡
Day 7	0.184±0.021	‡	‡
Day 8	0.222±0.048	‡	‡
Day 9	0.235±0.038	‡	‡
Day 10	0.257±0.037	‡	‡
Day 11	0.289±0.042	6.386±0.460	0.070±0.007
Day 12	0.324±0.044	8.148±1.045	0.090±0.006
Day 13	0.343±0.028	9.854±0.831	0.110±0.004
Day 14	0.407±0.053	11.670±1.024	0.154±0.012
Day 15	0.413±0.045	13.004±1.251	0.195±0.011
Day 16	0.435±0.046	14.168±1.180	0.270±0.010
Day 17	0.487±0.030	16.244±0.702	0.388±0.030
Day 18	0.499±0.022	17.242±0.810	0.407±0.020
Model Diagnostics			
n	60	40	40
R^2^	0.884	0.941	0.927
F value	443.680	605.220	481.600
P value	<0.001	<0.001	<0.001
Regression Equation	Y1 = −0.026+0.029X	Y2 = −10.480+1.557X	Y3 = −0.546+0.052X
Model diagnostics show linear regression parameters for incubation day (X) dependence of smooth muscle fractional anisotropy (Y1), length of tracked fibers (Y2) and FiberArea% (Y3) values.			

**Figure 2:**
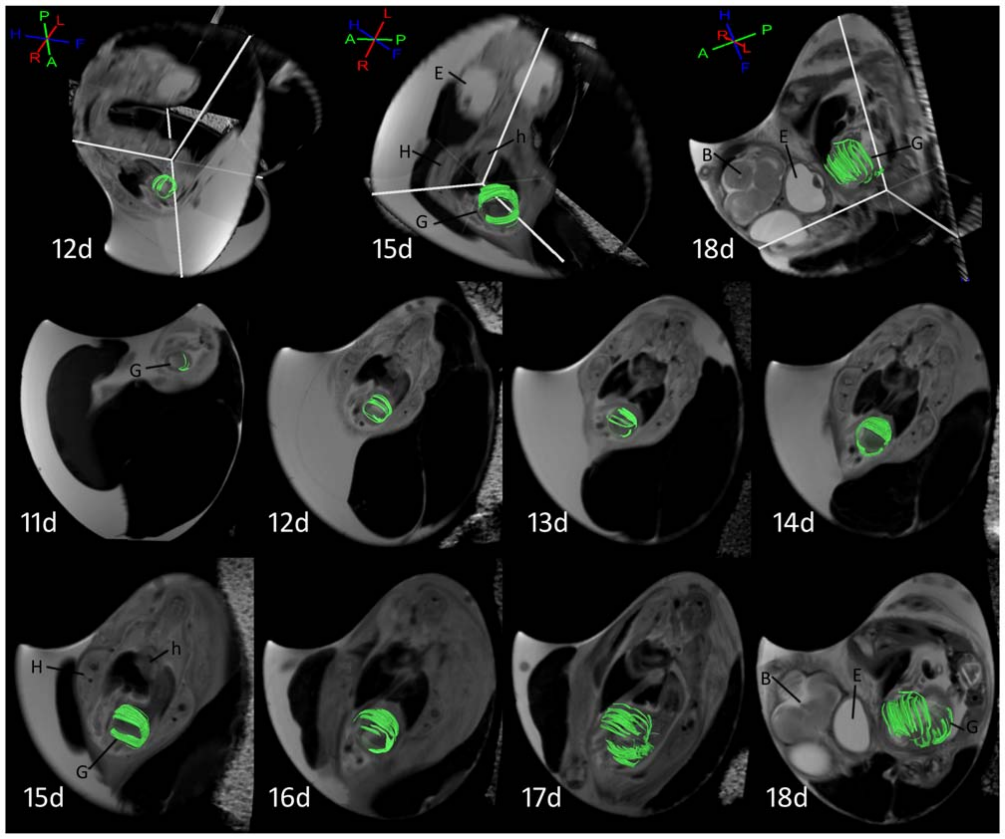
Serial smooth muscle fiber tracking result of embryonic gizzard from day 11 to day 18 of incubation. 3D images for days 12, 15, 18, with 3D axes provided in the upper left corner for orientation (*H:* head, *F:* foot, *R:* right, *L:* left, *P:* posterior, *A:* anterior). *Key*: *B*: brain, *E*: eye, *G*: gizzard, *H*: hind limb, *h:* heart.

**Figure 3:**
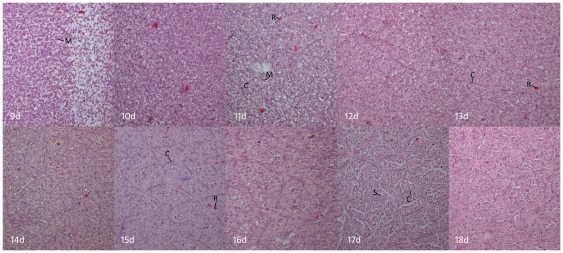
Histological specimens of chick embryonic gizzard at day 9 to day 18 of incubation. (200× magnification). Collagen bands and trellis-like muscle bundle confirmed at 11 day of incubation enlarges in subsequent days. *Key*: *C*: collagen band, *M*: myoblast, *R*: red blood cell, *S*: smooth muscle.

While fractional anisotropy values could be calculated via tissue diffusion measurements as early as day 7, diffusion-tensor tracking of muscle fibers was not successful until incubation day 11, which is essentially the same time that muscle fibers could be visualized on histologic specimens (days 10–11). Multiple linear regression analysis of FiberArea%, day of incubation (Day), smooth muscle fractional anisotropy (FA), fiber tract length (Length) from incubation days 11 through 18 is given in Table 2. Day and Length were found to be significant factors (p<0.05), and FA was found as nonsignificant (p = 0.7575) in consideration of the interparameter correlation. The simple correlation (zero order correlation coefficients) is in the order of Day, Length, and FA. The regression equation is Y(FiberArea%) = −0.7353+0.08225 X_1_(Day)-0.04275 X_2_(FA)-0.01869 X_3_(Length) (F-ratio 183.181, p<0.001, R^2^ = 0.9385) and the multiple correlation coefficient is 0.9688. Typical examples of DTI derived serial smooth muscle fiber tracking result are shown in Fig. 2, and show that smooth muscle fiber length and number increases with each successive day. Histologic specimens from incubation days 9 through 18 are shown in Fig. 3.

**Table 2:** Multiple linear regression among FiberArea%, Day, FA and Length.

Model Diagnostics			
Dependent variable (Y)	FiberArea%		
Independent variable (X)	Day of incubation(X1)	FA of ROI(X2)	Length of tracked fibers(X3)
Regression coefficient	0.0823	−0.0428	−0.0187
P value	<0.0001	0.7575	0.0131
Zero order correlation coefficients	0.9627	0.8471	0.9217
Regression equation	Y = −0.7353+0.0823 X1−0.0428 X2−0.0187 X3		
n	40		
R^2^	#0.9385		
F value	183.1807(P<0.001)		
Multiple correlation coefficient	0.9688		

## Discussion

Our data show that muscle fiber tracts can be discerned during the incubation period on diffusion tensor images when motion is sufficiently suppressed by cooling prior to and during imaging. In contrast to *ex vivo* analytical methods, which typically involve embryonic sacrifice, repeat observations can be performed on a single embryo throughout the incubation period.

Smooth muscle development of chick embryo gizzard has been previously elucidated [10]–[12]. At incubation day 6, the primordial gizzard consists of vascularized mesenchymal tissues and myoblasts which are actively dividing. At incubation day 7, thin myofilaments are associated with dense bodies that can be detected by electron microscopy. By day 9 of incubation, thick myofilaments are present within myoblasts, and the structural collagenous matrix which delineates myoblasts into muscle bundles begins to form. From day 10 of incubation until after hatching, myoblasts gradually elongate and develop into fully differentiated smooth muscle cells. The thickness of collagen bands which separate myoblasts into bundles continues to increase during this time. Our histologic analysis of sacrificed embryos is consistent with the literature, with marked elongation of myoblasts at incubation day 10 or 11 and tightly packed myofilaments at incubation day 18.

We were able to track smooth muscle fibers within the gizzard using diffusion tensor imaging on day 11, consistent with our histologic findings and findings discussed in the literature. Progressive fiber length elongation correlates with our histological quantification by multiple linear regression analysis, and shows diffusion fiber tracking in DTI to be a noninvasive method of monitoring smooth muscle fiber development. FA shows the degree of anisotropy (the isotropic mesenchymal tissues turn to anisotropic myoblasts or myofibers during development). Our histological analysis method (FiberArea%) depends on the enlargement of trellis-like muscle bundle separated by the collagen bands, and there is no linear correlation between muscle bundle enlargement and myoblast/myofiber anisotropy in our multiple regression analysis. Small samples (only 5 eggs for each day) and selected bias (only four ROIs were selected for FiberArea% calculation) may be a limitation and influence the results.

We believe that this method has the potential to allow noninvasive evaluation of other tract-like organizing tissue within avian embryos such as nerve fibers, though the number of diffusion directions would be increased (typically at least 20) and the subsequent increase in imaging time due the increase in the number directions while maintaining isotropic voxels would necessitate the use of a dedicated animal magnet. It is possible that developmental mutational abnormalities or injuries could be detected *in ovo* by diffusion tensor imaging. Diffusion tensor magnetic resonance imaging has been shown to reveal regional dysmyelination effects in the Shiverer mouse mutant, with diffusion-tensor acquired parameters correlating with histologic microanatomic changes [13]. Severely injured rodent spinal cords also showed quantitative changes in *in vivo* diffusion tensor imaging parameters correlating with histologic demyelination of injured regions [14]. We do not believe that our experimental techniques can be easily extended to allow sufficient embryonic motion suppression within mammalian embryos, though similar effects may be possible to measure within the developing chick embryo. Noninvasive monitoring of white matter tracts within the brain and spinal cord of chick embryos will be a subject for our future evaluation.

This study has several inherent limitations. Small embryonic structures, primarily during the early period of incubation, are difficult or impossible to evaluate due to the spatial resolution limitations of 3.0 T MRI; higher field strength magnets should improve spatial resolution. As in any study correlating *in vivo* imaging and *ex vivo* histology, acquiring exact matches between imaging planes and histologic specimens is technically challenging. Even with careful attention to detail, exact matching was not feasible in all cases. Chick embryo orientation and position within the egg naturally changes between successive days of imaging, and limits the ability to exactly match imaging planes between successive days. Despite this limitation, the proventriculus or gizzard could be consistently identified on magnetic resonance images after day 6. Lastly, while not apparent in our small sample, developmental differences may exist between embryos of the same hatching day.

Our study shows that diffusion tensor imaging of smooth muscle, using a widely-available 3.0 T magnetic resonance imaging system, holds promise as an investigative method for the serial *in vivo* evaluation of embryonic development. We show that tracked fiber length of smooth muscle in diffusion tensor imaging correlates with parameters derived from histologic specimens from sacrificed embryos. Noninvasive evaluation of development within embryonic models has predominantly consisted of anatomic evaluation. Diffusion tensor magnetic resonance imaging could provide a new method of understanding the mechanics of early development.

## Materials and Methods

### Animals and Treatments

The experimental protocol and procedures were approved by the Institutional Ethics Committee of the Shanghai Jiaotong University School of Medicine. Ninety (90) fertile Hy-Line White eggs each weighing 50–55 g were obtained from a commercial hatchery and placed in a digital tabletop incubator with temperature (37.8°C) and humidity (60%) controlled automatically. After four days of incubation, embryo development was evaluated by candling (using a hand-held light source, light was shone through the egg) to determine if they were fertile and developing normally. Eight eggs were removed from the incubator for underdevelopment. From the 82 remaining eggs, 5 eggs were chosen at random each day, from incubation days 5 to 18 (14 days total) and treated as follows: eggs were removed from the incubator and air-cooled for one hour at 3.5–4°C prior to imaging [5]. During imaging, the egg was wrapped in a single piece of Techni-Ice (Techni Ice, Victoria, Australia). Techni Ice surface (in contact with the egg) temperature was 0–2°C, measured immediately prior to and after imaging. Temperature monitoring during imaging was not performed. Embryonic motion was suppressed using the dual-cooling method. Total imaging duration was approximately 32 minutes. An additional 3 eggs were imaged serially without making histological specimen from day 5 to 18.

### MR Image Acquisition

Eggs wrapped in Techni-Ice were imaged in a 3.0 T Philips Achieva magnet (Philips Medical Systems, Best, Netherlands) using a four-channel dedicated animal coil with a 5 cm inner diameter. Image acquisition consisted of high-resolution T2 weighted TSE (turbo spin echo) images (accelerated with SENSE, TR/TE = 4375/80 ms, FOV 50×45×42 mm, ETL = 13, NEX = 10, matrix 250×225 (0.2×0.2 mm), slice thickness 1.2 mm, no gap, 12 min 23 s duration), and SENSE DTI using SE (spin echo)-DWI (15 directions, TR/TE = 5517/65 ms, FOV 50×45×42 mm, matrix = 83×75 (0.6×0.6 mm), b = 500 s/mm2, NEX = 2, slice thickness 1.2 mm, no gap, 20 minute duration). All imaging planes were sagittal. A 16^th^ unweighted (b = 0) DTI image was also acquired. Table 3 shows the 15 directions of DTI.

**Table 3:** The 15 Directions of DTI.

1, 0, 0
0, 1, 0
0, 0, 1
−0.1789, −0.1113, −0.9776
−0.0635, 0.3767, −0.9242
0.7108, 0.0516, −0.7015
0.6191, −0.4385, −0.6515
0.2424, 0.7843, −0.571
−0.2589, −0.618, −0.7423
−0.8169, 0.1697, −0.5513
−0.8438, 0.5261, −0.106
−0.2626, 0.9548, −0.1389
0.0001, 0.9689, 0.2476
0.7453, 0.6663, 0.0242
0.9726, 0.2317, 0.0209

### MR Fiber Tracking

**Figure 4:**
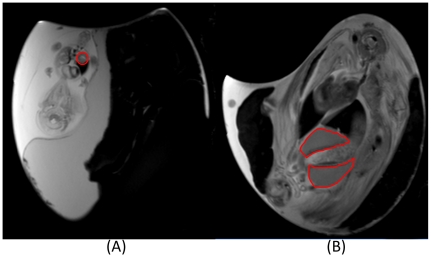
Region of interest (ROI) examples used for DTI fiber tracking. (**A**) At incubation day 9, a single ROI encompasses the entire proventriculus. (**B**) At incubation day 17, two ROIs each cover about half of the gizzard.

Smooth muscle fibers of the gizzard were tracked using FiberTrak software (Philips Medical Systems, Best, Netherlands). Diffusion registration was used prior to fiber tracking. Three parameters, minimum fractional anisotropy (FA), maximum angle change, and minimum fiber length were set to determine the stopping threshold of tractography. We chose a small minimum FA (0.15) and small maximum angle change (27°) to balance the sensitivity of smooth muscle fibers' tracking during early development stage and the precision during later development. A small minimum fiber length (5 mm) was used to enhance sensitivity during early development, with a longer minimum fiber length (10 mm) used during later development to avoid unrelated fibers from being tracked. High-resolution T2-weighted images were used to guide placement of the region of interest (ROI) required for seeding the fiber tracking algorithm. The sagittal image with the largest section of the gizzard was chosen for ROI placement. The ROI size changed with embryonic development; prior to day 11 of incubation, the ROI covered the entire proventriculus. After day 11 of incubation, two ROIs were used, each covering about half of the gizzard. Example ROIs are shown in Fig. 4.

### Histology

**Figure 5:**
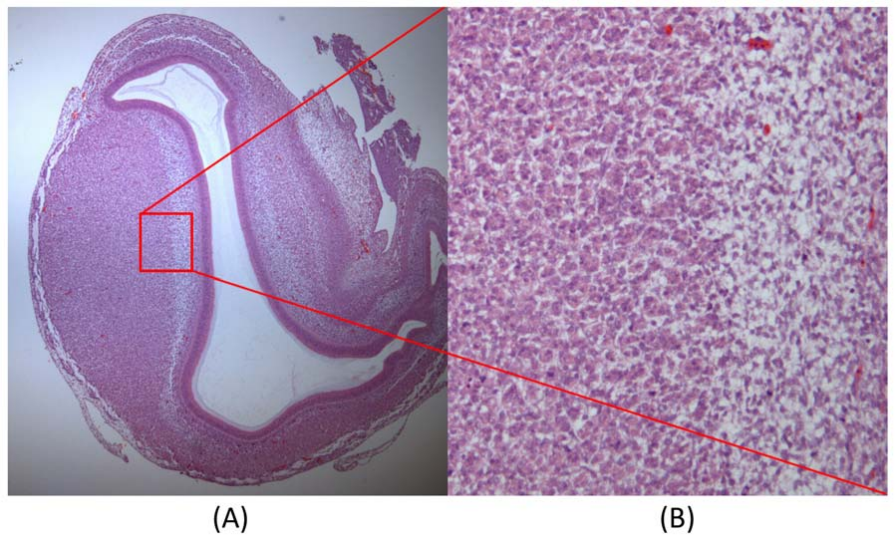
Example regions chosen for histologic sampling. (**A**) The chick embryonic gizzard at incubation day 9. (**B**) Micrograph (200× magnification, 1260×1400 resolution) of the red region of interest shown in (A).

After MR imaging, the egg was cracked and the corresponding embryonic proventriculus or gizzard was resected as a specimen and fixed in a 10% formaldehyde solution for one week. Following dehydration and paraffin embedding, the specimen was serially sectioned at 3 µm, in a plane corresponding to the sagittal MR imaging plane; sections were then stained with hematoxylin and eosin. Each specimen was evaluated with light microscopy (Olympus BX51, Olympus, Tokyo, Japan). The block region most closely corresponding to the DTI ROI was chosen and micrographed (200× magnification, 1260×1400 resolution; refer to Fig. 5).

### Data Analysis

**Figure 6:**
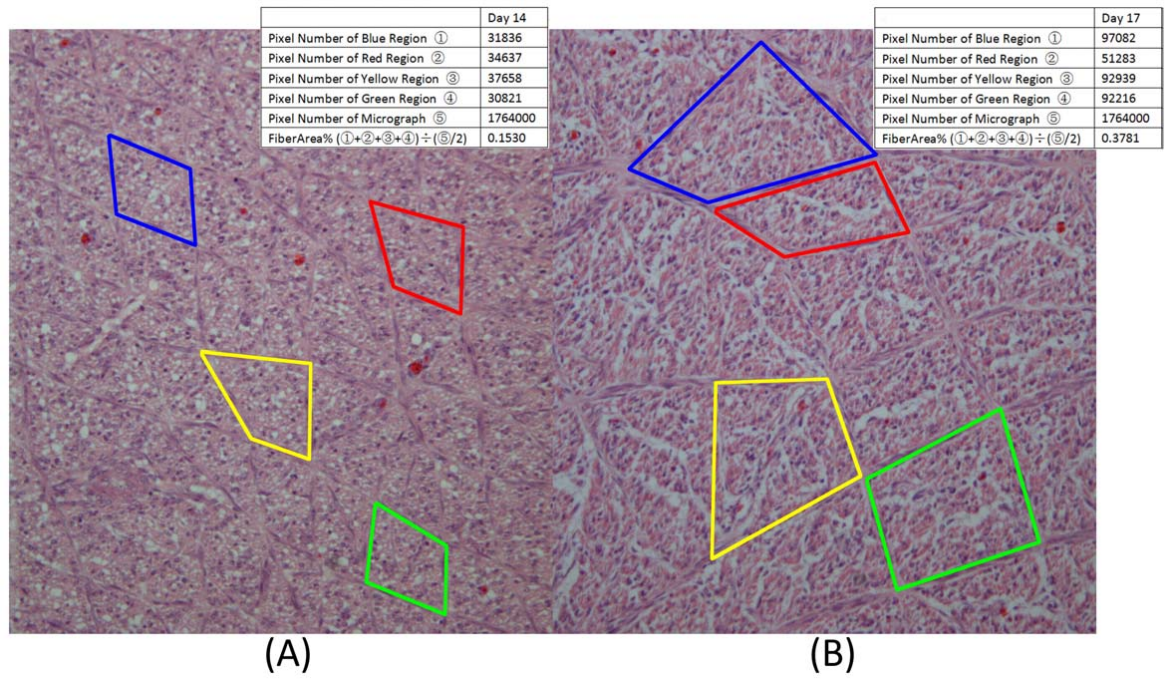
Depiction of the smooth muscle FiberArea% algorithm. Smooth muscle micrographs (200× magnification, 1260×1400 resolution) of embryonic gizzard at incubation day 14 (**A**) and incubation day 17 (**B**). Four muscle bundles are randomly chosen in each micrograph and the results of the FiberArea% algorithm are shown in right corner.

The fractional anisotropy (FA) value of tissue within ROI, an invariant scalar index which accounts for structural anisotropy in tissue, was automatically computed by the FiberTrak software. FA is defined as:
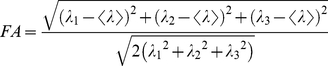
(1)where
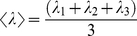
(2)λ_1_, λ_2_, and λ_3_ are the eigenvalues of the diagonalized diffusion tensor [8]. The Length of tracked fibers was also calculated by the software. Average smooth muscle fiber area density (termed FiberArea%) was determined using custom software based on the Insight Segmentation and Registration Toolkit (Kitware Inc., Clifton Park, NY). FiberArea% was calculated by selecting four randomly chosen muscle fiber bundles within the micrograph. The size (in pixels) of the micrograph is constant (1260×1400 resolution). Each muscle fiber bundle size, in pixels, was calculated. FiberArea% is the ratio of the area of the chosen muscle fiber bundles relative to the area of the micrograph. Examples at incubation day 14 and 17 of this method are shown in Figure 6.

Both statistical analysis and data management was performed using MedCalc (MedCalc Software, Mariakerke, Belgium). A linear model was used to evaluate the relationship between day of incubation and ROI-derived fractional anisotropy (FA) values, Length of tracked fibers, and FiberArea%. To evaluate the relationship between imaging and histology during embryonic development, the multiple regression analysis among FiberArea% (dependent), day of incubation (independent), FA of ROI (independent) and Length of tracked fibers (independent) was performed, with p values = 0.05 considered to be significant.
